# Correlates of Physical Activity, Psychosocial Factors, and Home Environment Exposure among U.S. Adolescents: Insights for Cancer Risk Reduction from the FLASHE Study

**DOI:** 10.3390/ijerph17165753

**Published:** 2020-08-09

**Authors:** Lei Xu, Charles R. Rogers, Tanya M. Halliday, Qiang Wu, Logan Wilmouth

**Affiliations:** 1 Department of Health Education and Promotion, East Carolina University, Greenville, NC 27834, USA; 2 Department of Family & Preventive Medicine, University of Utah School of Medicine, Salt Lake City, UT 84112, USA; Charles.Rogers@utah.edu; 3 Department of Health, Kinesiology, and Recreation, University of Utah, Salt Lake City, UT 84112, USA; tanya.halliday@utah.edu; 4 Department of Biostatistics, Brody School of Medicine, East Carolina University, Greenville, NC 27834, USA; WUQ@ecu.edu; 5 Department of Public Health, Brody School of Medicine, East Carolina University, Greenville, NC 27834, USA; wilmouthl15@students.ecu.edu

**Keywords:** adolescent, parent influence, home environment, physical activity

## Abstract

Background and aims: Physical activity (PA) can bring numerous health benefits to adolescents and can largely aid in reducing the various types of cancer risks in their lifespans. However, few adolescents meet the physical activity guidelines recommended by the National Cancer Institute in the United States. Our study aimed to examine the multilevel determinants potentially influencing adolescent’s PA participation. Methods: A secondary analysis of physical activity, home and school neighborhood, and other psychosocial data from 1504 dyads of adolescents and their parents who participated in the 2014 Family Life, Activity, Sun, Health, and Eating (FLASHE) study was performed. Analysis of variance and general linear model analyses were used to examine the correlates. Results: General linear modeling revealed that younger adolescents participated in greater levels of PA than older adolescents (*p* < 0.001). Adolescents whose parents reported meeting PA guidelines participated in greater amounts of PA (*p* < 0.001). Parental support of adolescent PA (*p* < 0.001) was also predictive of adolescent PA levels. Furthermore, parents who reported meeting moderate-to-vigorous physical activity (MVPA) guidelines were more likely to have teenagers that engaged in higher amounts of PA (*p* < 0.001). Discussion and Conclusions: Our findings imply a dynamic relationship between adolescent and parent MVPA levels. Interventions focused on increasing parental MVPA and encouraging parents to engage in promoting PA are merited in order to aid in increasing PA among adolescents while reducing the cancer risk.

## 1. Introduction

There is general consensus among health professionals that physical activity (PA) is conducive to disease prevention [1]. When established during adolescence and sustained throughout the lifespan, habitual PA may greatly contribute to the prevention of chronic disease, a number of types of cancers included [1]. According to the Centers for Disease Control and Prevention, being physically active can reduce an individual’s risk of developing bladder, endometrial, breast, colorectal, esophageal, kidney, lung, and stomach cancers [2]. Specifically, individuals who exercise regularly have a 20% to 50% lower risk of colorectal and breast cancers compared with those who do not exercise regularly [3].

Regrettably, the majority of adolescents do not meet current PA guidelines recommended by the national health authorities [3,4,5]. Less than 25% of adolescents in the United States (U.S.) are meeting the recommended amount of PA, i.e., 60 min or more of moderate-to-vigorous physical activity (MVPA) on a daily basis [6]. An insufficient engagement in MVPA places adolescents at higher risk for developing cancer later in life [2,3]. Even more concerning, adolescent PA levels declined over time [1]. The continuous decline of PA amongst adolescents has led to substantial research on potential facilitators and inhibitors that may influence adolescents’ PA levels, such as age, gender, familial/social support, peer influence, self-motivation, physical education, and environmental access to physical activities [4,7,8,9].

The determinants of PA participation among adolescents are complex and multifactorial. The multiple levels of influence—such as individual, interpersonal, organizational, community, and public policy—on adolescent PA can be partly explained through application of the socio-ecological model [10,11]. For instance, prior data has demonstrated that parent-level PA and parents’ socioeconomic status (SES) might impact adolescent PA [12,13,14,15,16,17]. Moreover, the neighborhood and rural or urban environment in which an adolescent resides can directly influence the amount of PA youths participate in [18]. It has been established that adolescents who reside in urban neighborhoods are more likely to participate in five or more hours a week of MVPA juxtaposed to their rural counterparts [19]. Additionally, the neighborhood environment in relation to the proximity to school influences how frequently students walk/bike to school [20]. In the 1960s, nearly 35% of adolescents lived within a mile of where they went to school; however, as of 2000, only 20% of adolescents did [21]. The percentage decline of adolescents living near their schools is directly correlated with a reduction in how many adolescents bike or walk daily [21]. 

However, a paucity of observational studies and interventions examining multi-determinants of PA in adolescents exist. It is also understudied how these multiple indicators correlate with cancer risk reductions and adolescent PA when employing a dyadic analysis among national samples. The Family Life, Activity, Sun, Health, and Eating (FLASHE) study resulted in a national dataset collected by the National Cancer Institute permitting this type of analysis [22]. The two-fold purpose of our current investigation was to utilize FLASHE to examine: (1) associations between sociodemographic factors, home/neighborhood environment, parenting patterns, and adolescent PA level and (2) predictors that influence the adolescent PA level to aid in decreasing cancer risks. We hypothesized that the parent MVPA level, parenting patterns towards PA, and adolescents’ home/neighborhood environments positively influenced the adolescent PA level. 

## 2. Methods

### 2.1. Design

FLASHE is a cross-sectional, internet-based observational study of parent/caregiver dyads that collected data on psychosocial, generational (parent-adolescent), and environmental correlates of cancer-preventive behaviors. A total of 1504 dyadic records were eligible for inclusion for the purpose of our study. The GeoFLASHE dataset was recently released to researchers as an extension of FLASHE and featured the examination of neighborhood characteristics, as well as parent-adolescent dietary and physical behaviors. The two key components of GeoFLASHE are the dyad’s home neighborhood and adolescents’ school neighborhood. This specific analysis was conducted in 2020 and focuses on the correlates of these psychosocial, environmental, and dyadic variables with adolescent MVPA [22]. Specifically, our outcome variable was total adolescent MVPA, and the independent variables were the adolescents’ ages, sex, race, body mass index (BMI) category, home/school rurality, home SES) quintile, the adolescents’ neighborhood environment, and parents’ reported attitudes towards teen PA, as well as the parental MVPA level. Detailed FLASHE design and methodology information has been published previously [22].

### 2.2. Sample 

A nonprobability sample was recruited from all U.S. regions. Based on sex, census division, household income and size, and race/ethnicity, eligible participants were balanced in the U.S. population. Within each household, one adolescent and one parent were selected from eligible household members. Parents were eligible if there were at least 18 years of age and lived with at least one child aged between 12 and 17 years of age for > 50% of the time. Adolescents were eligible if their ages were between 12–17 years old and lived in the household for > 50% of the time. Parents were informed regarding the study’s aim and procedures before their participation and approval of the participation of their adolescent(s) [23].

### 2.3. Procedures

Internet surveys were completed by each adolescent, asking them to self-report their age (12–13, 14–15, or 16–17 years); sex (male or female); and race/ethnicity (Non-Hispanic Black, Non-Hispanic White, and others), as well as height and weight. BMI categories (healthy weight, obese, overweight, and underweight) were determined from height, weight, and BMI percentiles. The FLASHE demographic survey asked parents to provide answers to two sets of open-ended questions about the location of their home and their adolescent’s school [22]. The home and school address responses were grouped into four categories: city, suburb, town, and rural. Neighborhood SES were measured based on the following domains: occupation, unemployment rate, poverty, income, education, and housing [24].

### 2.4. Variables

*Adolescent MVPA.* Total MVPA—including PA in and out of school—during both weekdays and weekends was determined via the previously validated Youth Activity Profile (YAP) and was used as the outcome variable in this data analysis. In brief, the YAP is a 15-item questionnaire developed for youths aged 9–18 years (4th through 12th grade) to provide general feedback regarding a student’s PA behavior. These PA behaviors were segmented into activities that occurred at school, out-of-school, and during weekends, in addition to the sedentary time taking place when out-of-school. YAP scores were calibrated into individual composite scores that allowed raw scores to be converted to an estimate of time spent per day in MVPA [25]. Teenagers were asked to answer five questions based on their PA levels during the last week and two questions regarding the levels of PA during the last weekend. For example, the questions included: (1) “How many days did you *WALK OR BIKE TO* a job, a friend’s house, or to an event or activity?”; (2) “How many days IN THE AFTERNOON (between 12:00pm/Noon-6:00 PM) did you do some form of physical activity for at least 10 minutes? This can include playing with your friends/family/supper camps, team practices, or classes involving a physical activity, but NOT walking or biking to a job, a friend’s house, or to an event or activity”; and (3) “How much physical activity did you do last SUNDAY? This could be for exercise, work/chores, family outing, sports, dance, or play?” [22]. This outcome variable was treated as continuous.

*Parent MVPA.* Parents completed a survey asking them to report all of the MVPA they completed in the past 7 days [24]. Parental PA was categorized as either meeting vs. not meeting national PA guidelines for adults of at least 75 min vigorous PA or 150 min moderate PA in a week or a combination of the two [3]. Parents who met one of the aforesaid criteria were considered as meeting the guidelines. Parents were asked to report all vigorous activities that they did in the past 7 days. Vigorous physical activities were defined as “activities that take hard physical effort and make you breathe much harder than normal and the duration for those physical activities lasted at least 10 min at a time”. For instance, parents were asked: (1) “During the LAST 7 DAYS, on how many days did you do VIGOROUS physical activities like heavy lifting, digging, aerobics, or fast bicycling?”(2)“How much time did you usually spend doing vigorous physical activities on one of those days?” (3) “During the LAST 7 DAYS, on how many days (per week) did you do MODERATE physical activity like carrying light loads, bicycling at a regular pace, or double tennis? Do not include walking.” [22].

*Adolescents’ home/neighborhood environment.* Parents were asked to indicate how much they disagree or agree with certain statements about their home neighborhood, which was defined as “the local area around home, within a 10-15-min walk in any direction.” These statements included: (1) “Many shops, stores, markets, or other places to buy things I need are within a 10-15-min walk of my home”; (2) “A transit stop like a bus, train, or trolley is within a 10–15-walk of my home”; (3) “There are sidewalks on most of the streets in my neighborhood”; (4) “My neighborhood has several or low-cost recreation facilities, such as parks, walking trails, bike paths, recreation centers, playgrounds, etc.”; (5) “The crime rate in my neighborhood makes it unsafe to go on walks at night; (6) People in this neighborhood help each other out”; (7) “We watch out for each other’s children in the neighborhood”; and (8) “There is litter or garbage on the streets or sidewalks in my neighborhood. Answer choices were “strongly disagree”, “somewhat disagree”, “somewhat agree”, and “strongly agree”, yet we recategorized these answers into two groups, i.e., “agree” and “disagree”” [22].

*Parental report on attitudes toward adolescents’ physical activity*. Parents were asked to provide their attitudes towards their adolescents’ PA, experiences at school, and time spent using or watching electronic devices such as laptops, smartphones, gaming systems, or televisions. Parents were reminded that PA includes any play, game, sport, exercise, or mode of transportation (like walking or biking to school) that gets adolescents moving and breathing harder. The statements included: (1) “I have to make sure my teenager gets enough physical activity”; (2) “ I take my teenager places where he/she can be physically active”; (3) “My teenager and I decide together how much physical activity he/she has to do”; (4) “I make my teenager exercise or go out and play”; (5) “I try to be physically active when my teenager is around”, and 6) “It is okay for me to make rules about how much time my teenager spends being physically active/playing. “Answers were “strongly disagree”, “somewhat disagree”, “neither disagree nor agree”, “somewhat agree”, and “strongly agree”, where we regrouped these answers into three categories, i.e., “agree”, “disagree”, or “neutral” [22]. 

### 2.5. Statistical Analysis

Summary statistics were calculated in frequencies and means (95% confidence limits). Two statistical analysis were used in examining the correlates in our data analysis process: analysis of variance (ANOVA) and general regression modeling. ANOVA tests were used to test bivariate relationships between adolescents’ PA levels and the demographic and independent variables. Those demographic and independent variables resulting in statistically significant bivariate relationships with adolescents’ PA levels were later entered into a general linear regression model to predict adolescents’ PA levels. A backward elimination method was employed to remove nonsignificant predictors from the model. The final model included only predictors with a *p*-value less than 0.05. Parameter estimates and related 95% confidence intervals were reported. All statistical tests used a significance level of 0.05. For our statistical analysis, SAS 9.4 (SAS Institute, Cary, NC, USA) was used.

### 2.6. Ethical Approval

Study procedures and protocol for the current study’s secondary data analyses were not required by the Institutional Review Board of East Carolina University. 

## 3. Results

The descriptive statistics and correlational analysis are presented in Table 1. The average MVPA of adolescents was 12.81 (95% CI = (12.68, 12.94)) h/week. The total dyadic sample contained 1678 adolescents (835 males and 843 females); however, only 1504 dyadic records were eligible for inclusion in this analysis due to the remainders containing missing data. Bivariate associations suggested that adolescents’ ages (*p* < 0.001) and parents’ MVPA levels (*p* < 0.001) were significantly associated with adolescents’ PA levels. In addition, adolescents’ home environments, specifically shops within a 10–15 min-walk of home (*p* < 0.001), transit stops within a 10–15-min walk of home (*p* = 0.013), sidewalks on most of the neighborhood streets (*p* = 0.022), and litter/garbage on the neighborhood streets or sidewalks (*p* = 0.029) were all significantly related to adolescents’ PA levels. All six items capturing parental patterns or attitudes towards their child’s participation in PA (*p* < 0.001 for all) were also associated with greater adolescent PA. None of the teens’ home/school locations, genders, ethnicities, BMI, or neighborhood SES were associated with the adolescents’ PA levels. 

Table 2 presents the general linear models, which show that adolescents’ ages (*p* < 0.001); parent MVPA levels (*p* < 0.001); neighborhood environments of many shops, stores, markets, or other places to buy things are within a 10–15-min walk of the teen’s home (*p* < 0.001); and parental patterns/attitudes, including “ I have to make sure my teenager gets enough physical activity (*p* = 0.001)”, “I take my teenager places where he/she can be physically active (*p* = 0.001) ”, and “I make my teenager exercise or go out and play (*p* = 0.008)” could predict adolescents’ PA levels. In comparison, caregivers who reported meeting MVPA levels were more likely to have teenagers with higher PA levels. Moreover, parents who agreed that “I have to make sure my teenager gets enough physical activity (*p* = 0.001)” agreed that “I take my teenager places where he/she can by physically active (*p* = 0.001)” and agreed that “I make my teenagers exercise or go out and play (*p* = 0.008)” could influence adolescents to have higher PA levels.

## 4. Discussion 

Physical inactivity continues to rise among adolescents in the U.S., as scientific evidence has consistently reported that a high percentage of adolescents do not participate in PA of any kind on a regular basis [6]. Our secondary analysis of an online dataset from a large, national sample of parents and their adolescents examined the associations between home/neighborhood environment, parents’ MVPA levels, parents’ perspectives on their adolescents’ PA, and adolescents’ overall PA levels. Our study revealed key factors that could influence adolescents’ PA and, ultimately, reduce cancer risks: being younger (12–15 vs. 16–17 years old), parents meeting adult MVPA guidelines, neighborhoods with convenience stores or places within 10~15 min walk of home, and parents that are supportive of adolescents’ PA habits. 

Our most salient finding was that parental attitudes and PA patterns could predict an adolescent’s PA level. A growing body of literature has identified that parents have a large influence on adolescent PA levels [14,15,26]. Parents have the potential to provide encouragement, modeling, and tangible support in order to promote PA to their child [12]. For instance, a recent analysis has revealed that a one-minute MVPA increase in parents results in a 0.21–to 0.24-min increase in MVPA in their children [13]. In agreement, parents’ self-reported MVPA levels in our study were significantly associated with their adolescents’ PA levels. Additionally, parents who made efforts to ensure their adolescents could have enough exercise and took their adolescents places she/he could be physically active or made their adolescents go out and be physically active could positively influence the PA of their children. Transporting their adolescents to places permitting them to be physically active, encouraging walking/biking to school, and limiting sedentary behaviors have been described as encouragement towards adolescents meeting MVPA recommendations, while concurrently reducing cancer risks and mortality [27,28]. Our study further affirms the importance of the parental role and highlights the importance of future interventions promoting protective behaviors—e.g., getting enough PA—through parental influence to reduce their children’s chances of future cancer developments. 

Secondly, our findings revealed that home neighborhood factors were significantly associated with adolescents’ PA levels. These factors included if there was access to shops, stores, markets, transit stops, or sidewalks around homes and neighborhoods. Safety features in neighborhoods such as pedestrian lights, sidewalks, and traffic lights have proven to increase the likelihood of adolescents walking/biking to and from neighborhood destinations [20]. In a recent FLASHE study exploring neighborhood food environments and PA among U.S. adolescents, Johnson et al. [6] reiterated how increasing access to various retail food destinations within walking distance could possibly provide more PA for adolescents. Our study corroborates these findings and provides further support of the prospective impacts of expanding convenience shops, transit stops, or other walkable facilities to increase adolescent PA. 

Next, the results from our FLASHE study indicated that younger adolescents engaged in higher levels of PA than older youths (16–17 years of age). This finding is in agreement with former research establishing that PA declines during the adolescent period [29,30]. From a health benefit perspective, it is well-known that the positive health benefits of being physically active could be carried forward into later stages of teenagers’ lives [2]. For instance, girls and female adolescents who participate in 7 h/week of PA between ages 5–19 may reduce their breast cancer risk due to the breast development and hormonal changes during this age window [31]. PA among adolescents may also facilitate the prevention of diabetes [32]—a risk factor for cardiovascular disease and several cancers, including bladder, colon, liver, endometrial, and pancreatic [2]. Since at least 60 min of MVPA/day is recommended by the U.S. Department of Health and Human Services for youths aged 6–17 [33], interventions that result in children and adolescents obtaining and maintaining adequate levels of PA throughout development would potentially decrease the prevalence of chronic health conditions in young adults significantly. However, prior research has shown that adolescents are generally not concerned with chronic adverse health outcomes, but they could be motivated to engage in PA based on an increased awareness of the immediate health benefits [34,35,36]. While interventions aimed at the maintenance of PA during the early teenage years are imperative, highlighting the value of lifetime PA on chronic conditions, such as a risk reduction for diabetes and cancer, may not be a fitting motivator. Hence, future interventions should focus on immediate outcomes (self-esteem, energy, body image, mental health, etc.) that adolescents may find more motivating.

Lastly, we did not uncover correlations between teens’ home locations, school locations, neighborhood SES, adolescents’ genders, and adolescents’ BMI with adolescents’ levels of PA. Our findings diverged from previous studies, which have found that adolescents with a higher SES are more active than adolescents with lower SES [16]. It has been concluded that the differences in inclination towards PA among adolescents is directly related to economic factors [17]. Adolescents with higher SES often participate in activities requiring financial resources (e.g., gear, membership, and transportation) [16,37,38]. While hypothetical, it is conceivable that our results differed from prior works in this area due to the recruitment methods utilized by the FLASHE team. By way of a convenient sample being used, it is likely that adolescent-caregiver dyads interested in this study were comprised primarily of adolescents with relatively high PA, as shown by the high level of self-reported PA. Likewise, it is also likely that PA was over-reported. Without sufficient spread, we were unable to detect differences in adolescents’ PA by SES, home location, gender, or BMI category [12,13,14,15,16,17].

### Strengths and Limitations

While the FLASHE and GeoFLASHE datasets provide information on cancer risk reduction behaviors from a large, regionally diverse sample of the U.S. population, as well as neighborhood environmental information, there are limitations to be acknowledged. First, a nonprobability convenience sample was recruited, which decreased the ability to generalize the results to the broader U.S. population. However, in an effort to ensure a representative participant pool, the FLASHE study team did balance recruitment based on U.S. population sex, census division, household income, and size, plus race/ethnicity. Second, due to the analytic procedures (coding participants into two or three groups based on their categorical responses to survey questions), the unequal sample sizes within these groups could be a limitation. However, our analytical plan was robustly considerate of differences in the sample sizes. Third, all anthropometric and PA data were collected via self-reporting and, therefore, was subject to biases that potentially make them inaccurate. Future trials that directly measure PA among parent-adolescent dyads via actigraphy and determine BMI via researcher-measured height and weight would strengthen the findings. Fourth, PA is only one aspect of cancer risk reduction, and many other lifestyle factors interact to determine the risk. Yet, the focus of our study was to test correlations and predicators among factors such as the home environment and parents’ attitudes that may influence adolescent PA levels. Lastly, the majority (72.5%) of FLASHE caregivers were women [22]. Therefore, the results on the influence of parental PA on adolescent PA cannot be extended to male caregivers, but future research in this area is warranted.

## 5. Conclusions

Using a nationally representative dataset, our study provided information on factors that influence adolescent PA uptake. Multiple determinants predicting adolescent PA behaviors were identified. Most notably, adolescents were more physically active when they had parents who met adult PA recommendations themselves and/or provided support to ensure the completion of physical activities among their adolescent children. Highlighting parents’ significant roles in their adolescent children’s’ PA levels is a critical component for future behavior change-focused interventions among adolescents. Moreover, multidimensional interventions that promote adolescents’ PA levels should be integrated into existing programs for improved health outcomes and reduced cancer risks.

## Figures and Tables

**Table 1:** Summary of the sample and the bivariate association with adolescents’ physical activity (PA) levels (total hours of PA in school, out-of-school, and on weekends).

Subgroup	Category	Total (N)	Mean	Standard Error	95% Confidence Limits		*p*-Value
Entire sample	All	1504	12.81	0.06	12.68	12.94	
Adolescent Age	12–13 years old	190	15.81	0.12	15.57	16.05	**<0.001**
	14–15 years old	557	14.12	0.07	13.98	14.26	
	16–17 years old	580	11.51	0.07	11.37	11.65	
Adolescent Gender	Male	734	12.85	0.10	12.66	13.04	0.599
	Female	765	12.78	0.09	12.60	12.95	
Adolescent Ethnicity	Non-Hispanic White	952	12.79	0.08	12.63	12.94	0.527
	Non-Hispanic Black	250	12.75	0.17	12.43	13.08	
	Hispanic	148	13.11	0.19	12.73	13.48	
	Other	138	12.81	0.26	12.30	13.32	
Adolescent BMI	Healthy weight	1014	12.79	0.08	12.64	12.94	0.165
	Obese	175	12.52	0.21	12.11	12.94	
	Overweight	215	12.99	0.17	12.65	13.33	
	Underweight	100	13.13	0.26	12.62	13.65	
Adolescent Home Location	City	393	12.97	0.13	12.71	12.94	0.402
	Suburb	619	12.74	0.10	12.55	13.22	
	Town	128	12.59	0.23	12.14	13.05	
	Rural	293	12.80	0.14	12.52	13.09	
Adolescent School Location	City	378	12.85	0.13	12.60	13.10	0.939
	Suburb	559	12.81	0.11	12.60	13.02	
	Town	145	12.86	0.20	12.47	13.26	
	Rural	233	12.73	0.17	12.40	13.07	
Neighborhood SES	Low	229	12.72	0.17	12.39	13.06	
	Med-Low	287	13.13	0.15	12.83	13.43	0.063
	Median	308	12.53	0.15	12.24	12.82	
	Med-High	323	12.83	0.13	12.57	13.09	
	High	280	12.82	0.14	12.54	13.10	
Parents’ MVPA level	Meet guideline	720	13.05	0.09	12.87	13.23	**<0.001**
	Not meet guideline	558	12.40	0.11	12.19	12.62	
Adolescents’ home/neighborhood environments							
Many shops, stores, markets, or other places to buy things I need are within a 10–15-minute walk of my home.	Disagree	874	12.62	0.09	12.45	12.78	**<0.001**
	Agree	615	13.09	0.10	12.90	13.29	
A transit stop like a bus, train, or trolley is within a 10–15-walk of my home.	Disagree	866	12.66	0.08	12.50	12.83	**0.013**
	Agree	618	13.00	0.10	12.79	13.20	
There are sidewalks on most of the streets in my neighborhood.	Disagree	548	12.62	0.11	12.41	12.83	**0.022**
	Agree	933	12.93	0.08	12.77	13.09	
My neighborhood has several FREE or low-cost recreation facilities, such as parks, walking trails, bike paths, recreation centers, playgrounds, etc.	Disagree	576	12.70	0.11	12.49	12.91	0.183
	Agree	911	12.88	0.08	12.72	13.04	
The crime rate in my neighborhood makes it unsafe to go on walks at night.	Disagree	1107	12.81	0.07	12.67	12.95	0.962
	Agree	383	12.80	0.14	12.52	13.08	
People in this neighborhood help each other out.	Disagree	414	12.64	0.13	12.38	12.89	0.103
	Agree	1075	12.88	0.08	12.73	13.02	
We watch out for each other’s children in the neighborhood.	Disagree	402	12.63	0.13	12.38	12.88	0.085
	Agree	1081	12.88	0.08	12.73	13.03	
There is litter or garbage on the streets or sidewalks in my neighborhood.	Disagree	1151	12.73	0.07	12.59	12.87	**0.029**
	Agree	339	13.07	0.15	12.77	13.37	
Parental patterns or attitudes towards adolescents’ physical activity							
I have to make sure my teenager gets enough physical activity.	Disagree	436	12.76	0.12	12.53	12.99	**0.001**
	Neutral	240	12.31	0.16	11.99	12.64	
	Agree	810	13.00	0.09	12.82	13.17	
I take my teenager places where he/she can be physically active.	Disagree	259	11.92	0.16	11.60	12.23	**<0.001**
	Neutral	270	12.35	0.16	12.03	12.66	
	Agree	956	13.19	0.08	13.04	13.34	
My teenager and I decide together how much physical activity he/she has to do.	Disagree	592	12.36	0.10	12.16	12.56	**<0.001**
	Neutral	413	12.88	0.12	12.63	13.12	
	Agree	480	13.32	0.11	13.10	13.54	
I make my teenager exercise or go out and play.	Disagree	548	12.21	0.11	12.00	12.42	**<0.001**
	Neutral	267	12.50	0.15	12.20	12.79	
	Agree	670	13.43	0.09	13.24	13.61	
I try to be physically active when my teenager is around.	Disagree	284	12.38	0.15	12.09	12.68	**<0.001**
	Neutral	384	12.55	0.13	12.29	12.81	
	Agree	817	13.09	0.09	12.93	13.26	
It is okay for me to make rules about how much time my teenager does physical activity.	Disagree	142	12.01	0.20	11.60	12.41	**<0.001**
	Neutral	428	12.43	0.12	12.19	12.67	
	Agree	914	13.11	0.08	12.95	13.27	

Note: BMI: body mass index, SES: socioeconomic status, and MVPA: moderate-to-vigorous physical activity. Boldface indicates statistical significance (*p* < 0.05).

**Table 2:** A general linear model predicting adolescents’ physical activity levels (total hours of PA in school, out-of-school, and on weekends).

Predictor	Category	Estimate	Standard Error	95% Confidence Limits		*p*-Value
Adolescent age	(12–13 vs. 16–17)	4.42	0.15	4.13	4.72	**<0.001**
	(14–15 vs. 16–17)	2.54	0.11	2.33	2.75	
Parent MVPA level	(Meet vs. Not meet guideline)	0.56	0.10	0.36	0.76	**<0.001**
Many shops, stores, markets, or other places to buy things I need are within a 10–15-min walk of my home.	(Agree vs. Disagree)	0.36	0.10	0.16	0.56	**<0.001**
A transit stop like a bus, train, or trolley is within a 10–15-walk of my home.	(Agree vs. Disagree)	−0.12	0.11	−0.35	0.10	0.284
There is litter or garbage on the streets or sidewalks in my neighborhood.	(Agree vs. Disagree)	−0.07	0.12	−0.31	0.16	0.540
I have to make sure my teenager gets enough physical activity.	(Neutral vs. Disagree)	−0.32	0.16	−0.63	−0.01	**0.001**
	(Agree vs. Disagree)	−0.50	0.13	−0.74	−0.25	
My teenager and I decide together how much physical activity he/she has to do.	(Neutral vs. Disagree)	0.10	0.14	−0.18	0.37	0.490
	(Agree vs. Disagree)	0.09	0.15	−0.55	0.38	
I take my teenager places where he/she can by physically active.	(Neutral vs. Disagree)	0.21	0.17	−0.12	0.55	**0.001**
	(Agree vs. Disagree)	0.50	0.14	0.22	0.79	
I make my teenager exercise or go out and play.	(Neutral vs. Disagree)	−0.18	0.15	−0.47	0.12	**0.008**
	(Agree vs. Disagree)	0.26	0.13	0.01	0.51	
I try to be physically active when my teenager is around.	(Neutral vs. Disagree)	0.06	0.16	−0.25	0.37	0.927
	(Agree vs. Disagree)	0.04	0.16	−0.27	0.34	
It is okay for me to make rules about how much time my teenager does physical activity.	(Neutral vs. Disagree)	−0.05	0.20	−0.45	0.34	0.622
	(Agree vs. Disagree)	−0.15	0.20	−053	0.34	

Note: Boldface indicates statistical significance (*p* < 0.05).
